# A Semiautonomous Deep Learning System to Reduce False Positives in Screening Mammography

**DOI:** 10.1148/ryai.230033

**Published:** 2024-04-10

**Authors:** Stefano Pedemonte, Trevor Tsue, Brent Mombourquette, Yen Nhi Truong Vu, Thomas Matthews, Rodrigo Morales Hoil, Meet Shah, Nikita Ghare, Naomi Zingman-Daniels, Susan Holley, Catherine M. Appleton, Jason Su, Richard L. Wahl

**Affiliations:** From Whiterabbit.ai, 3930 Freedom Cir, Santa Clara, CA 95054 (S.P., T.T., B.M., Y.N.T.V., T.M., R.M.H., M.S., N.G., N.Z.D., J.S.); Onsite Women's Health, Westfield, Mass (S.H.); SSM Health, St Louis, Mo (C.M.A.); and Mallinckrodt Institute of Radiology, Washington University School of Medicine, St Louis, Mo (R.L.W.).

**Keywords:** Artificial Intelligence, Semiautonomous Deep Learning, Breast Cancer, Screening Mammograp

## Abstract

**Purpose:**

To evaluate the ability of a semiautonomous artificial intelligence (AI) model to identify screening mammograms not suspicious for breast cancer and reduce the number of false-positive examinations.

**Materials and Methods:**

The deep learning algorithm was trained using 123 248 two-dimensional digital mammograms (6161 cancers) and a retrospective study was performed on three nonoverlapping datasets of 14 831 screening mammography examinations (1026 cancers) from two U.S. institutions and one U.K. institution (2008–2017). The stand-alone performance of humans and AI was compared. Human plus AI performance was simulated to examine reductions in the cancer detection rate, number of examinations, false-positive callbacks, and benign biopsies. Metrics were adjusted to mimic the natural distribution of a screening population, and bootstrapped CIs and *P* values were calculated.

**Results:**

Retrospective evaluation on all datasets showed minimal changes to the cancer detection rate with use of the AI device (noninferiority margin of 0.25 cancers per 1000 examinations: U.S. dataset 1, *P* = .02; U.S. dataset 2, *P* < .001; U.K. dataset, *P* < .001). On U.S. dataset 1 (11 592 mammograms; 101 cancers; 3810 female patients; mean age, 57.3 years ± 10.0 [SD]), the device reduced screening examinations requiring radiologist interpretation by 41.6% (95% CI: 40.6%, 42.4%; *P* < .001), diagnostic examinations callbacks by 31.1% (95% CI: 28.7%, 33.4%; *P* < .001), and benign needle biopsies by 7.4% (95% CI: 4.1%, 12.4%; *P* < .001). U.S. dataset 2 (1362 mammograms; 330 cancers; 1293 female patients; mean age, 55.4 years ± 10.5) was reduced by 19.5% (95% CI: 16.9%, 22.1%; *P* < .001), 11.9% (95% CI: 8.6%, 15.7%; *P* < .001), and 6.5% (95% CI: 0.0%, 19.0%; *P* = .08), respectively. The U.K. dataset (1877 mammograms; 595 cancers; 1491 female patients; mean age, 63.5 years ± 7.1) was reduced by 36.8% (95% CI: 34.4%, 39.7%; *P* < .001), 17.1% (95% CI: 5.9%, 30.1%: *P* < .001), and 5.9% (95% CI: 2.9%, 11.5%; *P* < .001), respectively.

**Conclusion:**

This work demonstrates the potential of a semiautonomous breast cancer screening system to reduce false positives, unnecessary procedures, patient anxiety, and medical expenses.

**Keywords:** Artificial Intelligence, Semiautonomous Deep Learning, Breast Cancer, Screening Mammography

*Supplemental material is available for this article.*

Published under a CC BY 4.0 license.

## Graphical abstract

**Figure:**
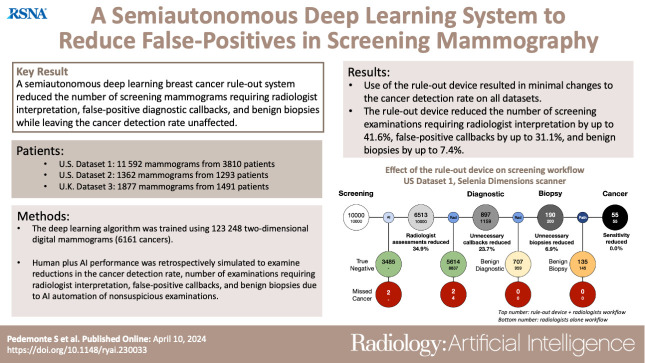

**Summary**

In a retrospective simulation study, a semiautonomous deep learning breast cancer rule-out system reduced the number of screening mammograms requiring radiologist interpretation, false-positive diagnostic callbacks, and benign biopsies while leaving the cancer detection rate unaffected.

**Key Points**

■ The semiautonomous artificial intelligence (AI) breast cancer screening model reduced the number of screening mammographic examinations requiring radiologist interpretation (U.S. dataset 1, 41.6% [95% CI: 40.6%, 42.4%]; *P* < .001; U.S. dataset 2, 19.5% [95% CI: 16.9%, 22.1%]; *P* < .001; U.K. dataset 3, 36.8% [95% CI: 34.4%, 39.7%]; *P* < .001) with minimal effect to sensitivity (U.S. dataset 1, *P* = .02; U.S. dataset 2, *P* < .001; U.K. dataset 3, *P* < .001).
■ A new labeling scheme was introduced to analyze the downstream impact of the human plus AI paradigm in a clinical workflow simulation. In addition to reducing the number of examinations requiring radiologist interpretation, the rule-out system also reduced the number of false-positive callbacks (U.S. dataset 1, 31.1%; *P* < .001; U.S. dataset 2, 11.9%; *P* < .001; U.K. dataset 3, 17.1%; *P* < .001) and benign biopsies (U.S. dataset 1, 7.4%; *P* < .001; U.S. dataset 2, 6.5%; *P* = .08; U.K. dataset 3, 5.9%; *P* < .001).

## Introduction

Globally, breast cancer is the most common cancer among female individuals and is predicted to result in the most cancer deaths for this population (1). Screening mammograms allow early detection, improve prognosis, and reduce mortality (2–6). Many nations have developed screening programs (7,8); the United States alone performs more than 38 million examinations yearly (9,10). False positives are a concern, as over 50% of individuals undergoing 10 screening examinations will experience false-positive callbacks and over 20% will undergo unnecessary biopsies (10,11). These false positives result in unnecessary diagnostic examinations, invasive diagnostic procedures, and patient anxiety (12,13). False positives constitute a substantial expenditure for health care systems, approximately $2.8 billion annually in the United States (14,15). To mitigate these harms, in 2009, the U.S. Preventive Services Task Force (USPSTF) recommended changing from annual screening starting at 40 years of age to biennial screening starting at age 50 years of age (16). However, recent updated recommendations suggest screening should begin at 40 years of age.

Most existing computer-aided detection and diagnosis software for cancer screening attempt to balance sensitivity and specificity by having roughly equal rates of false positives and false negatives. For example, computer-aided triage (17,18), computer-aided detection (17,19), and automated second interpretation (17,18,20) in double-reading settings (eg, United Kingdom and Europe) (21) have been implemented. These systems use an underlying algorithm that balances sensitivity and specificity at around 85% (22). This operating point may not be appropriate for workflows in which the goal is to automate the interpretation of nonsuspicious examinations rather than highlight suspicious findings. Unlike the assistive setting of computer-aided detection devices, autonomous workflows do not allow radiologists to correct the false-negative errors of the device.

Devices that rule out cancer are an emerging paradigm in screening (23–25). Rule-out devices operate at an extreme point of high sensitivity, near 100%. This operating point ensures that nearly all cases marked as nonsuspicious are truly cancer free and can be removed from the radiologist's workflow. Previous works have proposed rule-out devices that can automatically declare 17.0% (23), 19.3% (24), 60.0% (25), 34.3% (26), and 30.9% (27) of the mammograms as nonsuspicious with a sensitivity of 99.0%, 99.0%, 80.1%, 99.0%, and 97.8%, respectively. These findings suggest that such devices can potentially automate a portion of examinations.

In this study, we evaluated the ability of a breast cancer rule-out device to automate a large fraction of screening mammography examinations and reduce the number of false-positive callbacks and biopsies in simulations performed on large retrospective datasets from the United States and United Kingdom. We introduced a new labeling scheme to analyze how rule-out devices affect radiologist workflow and performance in the human plus artificial intelligence (AI) paradigm. This scheme allows us to highlight potential downstream benefits of rule-out devices, such as the reduction of invasive biopsy procedures for patients without cancer.

## Materials and Methods

This study used retrospective anonymized data to train and evaluate a deep learning system that identifies mammograms not suspicious for breast cancer. The objective was to assess the potential impact of a rule-out device on radiologists’ workflows for screening examinations. This study was approved by the relevant institutional review boards for anonymized data. Informed consent was waived, and data were handled according to the Health Insurance Portability and Accountability Act. This work was supported by funding from Whiterabbit.ai. Washington University in St Louis has equity interests in Whiterabbit.ai and may receive royalty income and milestone payments according to an agreement with Whiterabbit.ai to develop the technology in this research. The inclusion of data and analyses was controlled by R.L.W., who is not an employee or consultant of Whiterabbit.ai.

### Data

**Figure 1:**
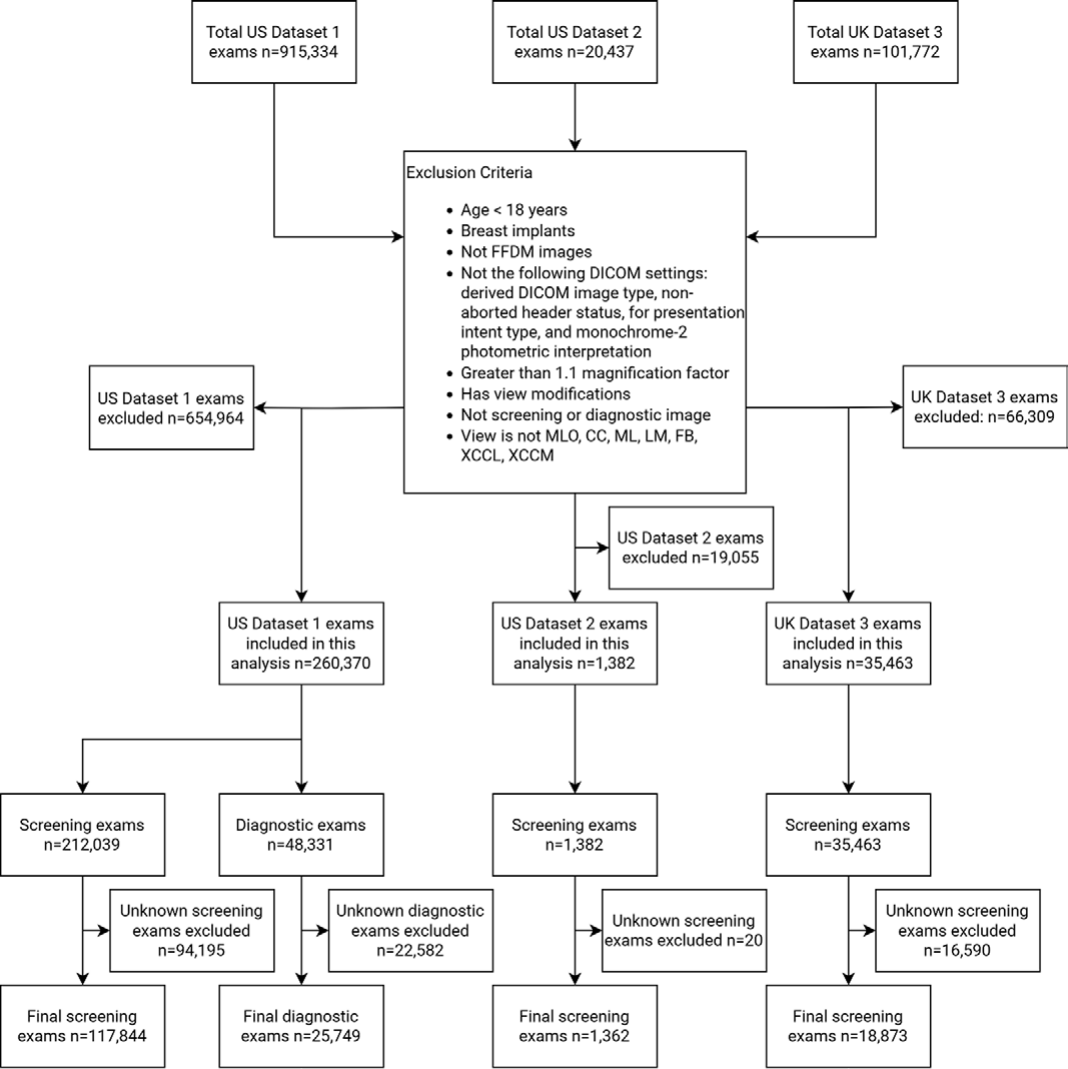
Flow diagram for the exclusion of mammography examinations for U.S. dataset 1, U.S. dataset 2, and U.K. dataset 3. Only U.S. dataset 1 had diagnostic examinations which were used only during model development to increase the number of cancers available for training. CC = craniocaudal, DICOM = Digital Imaging and Communications in Medicine, FB = from below, FFDM = full-field digital mammography, LM = lateromedial, ML = mediolateral, MLO = mediolateral oblique, XCCL = exaggerated craniocaudal laterally, XCCM = exaggerated craniocaudal medially.

Two-dimensional full-field digital mammography examinations were gathered from three institutions: two U.S. institutions (U.S. dataset 1 from 2008 through 2017 and U.S. dataset 2 from 2014 through 2019) and one U.K. institution (U.K. dataset 3 from 2011 through 2015) (28). Figure 1 shows the exclusion criteria.

U.S. dataset 1 and U.K. dataset 3 were randomly divided into three nonoverlapping datasets by patients (Table 1): a training set (80%), a validation set for tuning hyperparameters and selecting operating points (10%), and a test set (10%). U.S. dataset 2 was completely held out for testing. Mammograms were acquired using Hologic Selenia (HSE) and Hologic Selenia Dimensions (SED) scanner models.

**Table 1:**
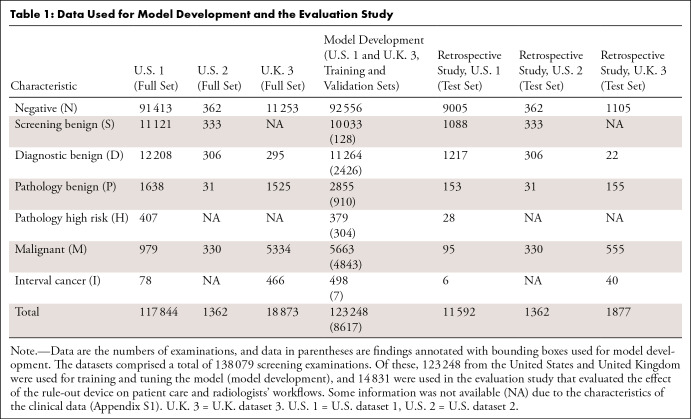
Data Used for Model Development and the Evaluation Study

U.S. dataset 1 was interpreted by 28 breast radiologists with experience ranging from 2 to 30 years from 2008 to 2017 (average number of reads, 392.8 ± 602.2 [SD]; range, 1–1765). U.S. dataset 2 was interpreted by 59 radiologists, 16 of whom were fellowship trained, with experience ranging from 1 to 37 years from 2014 to 2019 (average number of reads, 24.7 ± 37.5; range, 1–190). U.K. dataset 3 was interpreted by 210 readers of unknown experience levels from 2011 to 2015 (average number of reads, 24.0 ± 47.0; range, 1–270).

Labels were assigned to each breast based on biopsy outcomes and the assessments of the original reporting radiologists (Table 1): negative (N) for Breast Imaging Reporting and Data System (BI-RADS) 1, screening benign (S) for BI-RADS 2, diagnostic benign (D) for BI-RADS 0 followed by a negative diagnostic assessment, pathology benign (P) for benign pathology assessments, high risk (H) for nonupstaged high-risk pathology assessments, malignant (M) for malignant pathology assessments, and interval cancers (I) for a BI-RADS 1 or 2 with a malignant pathology assessment within the screening interval (12 months for the U.S. dataset and 36 months for the U.K. dataset) and prior to the subsequent screening examination (Appendix S1). The N, S, and D labels needed a follow-up examination at least 2 years later and no biopsy events in the entire patient history. The remaining breasts were labeled unknown (U) and were excluded. Labels were propagated to examinations by selecting the maximum outcome from the two breasts from N (lowest), S, D, U, P, H, I, to M (highest). Since the United Kingdom does not use the BI-RADS lexicon, these labels were generated given the reader opinions. Screening examinations with a final opinion of normal or benign were labeled BI-RADS 1 or BI-RADS 2, respectively. Screening examinations with a final reader's opinion other than normal or benign were labeled BI-RADS 0. Other examinations with nonscreening opinions of benign, suspicious, or uncertain, or malignant were labeled BI-RADS 3, 4, or 5, respectively.

### Outcomes

Biopsy-ascertained outcomes were used as the reference standard. It was determined whether the patient developed cancer within a time window from the screening examination. Windows of 6, 12, and 24 months were employed for the U.S. datasets, and 6, 12, 24, of 36 months were employed for the U.K. dataset. The cancer-positive class contained the M and I outcomes. The negative class contained the examinations with outcomes N, S, D, P, and H (Appendix S7).

### Development of the Rule-out Algorithm

The algorithm is composed of a low-level vision (deep learning) model that analyzes each image independently and a high-level vision model (metamodel) that combines the low-level information to compute a final examination malignancy probability. This architecture enables the algorithm to (*a*) utilize multiview, bilateral, and prior imaging data and (*b*) integrate imaging and nonimaging information. The cancer detection algorithm operates on four inputs: the mammogram images, the patient's age, the prior mammogram's images, and the BI-RADS assessments for prior examinations where available. More information on the usage of the training and validation datasets for model development of the individual low-level vision and high-level vision models can be found in Appendix S2.

### Rule-out Workflow and Operating Point Selection

To simulate the rule-out workflow in which the device reads the examinations before radiologists, examinations with predictions lower than the rule-out operating threshold are assigned a negative prediction (BI-RADS 1). For all other examinations, the original clinical assessment is assigned, modeling the scenario in which radiologists’ interpretations are unaffected by the fact that the device did not rule out the examination. After training, we calculated the rule-out threshold using the U.S. dataset 1 and U.K. dataset 3 validation datasets. The threshold was calculated to achieve a 12-month prediction target cancer sensitivity of 99% and 97% (Appendix S4).

### Metrics

We characterized the radiologists and device plus radiologists system using the cancer detection rate (CDR), false-positive callback reduction rate, and benign biopsy reduction rate. We characterized the stand-alone device by the absolute and relative sensitivity, rule-out rate, reduction of false-positive callbacks, and reduction of benign biopsies. The radiologists’ true positives were cancers with BI-RADS 0, 4, 5, and 6 assessments. The device's true positives were the cancers with predicted scores greater than or equal to the rule-out operating threshold. The device plus radiologists true positives were cancers with BI-RADS 0, 4, 5, and 6 assessments and a device score greater than or equal to the rule-out operating threshold. The absolute device sensitivity was the device's true positives over the total number of cancers. The relative device sensitivity was the intersection between the device's true positives and radiologists’ true positives over the radiologists’ true positives (Appendix S5). The rule-out rate was the percent of screening examinations with a score less than the rule-out operating threshold.

### Statistical Analysis

Performance is reported on the test datasets of U.S. dataset 1, U.S. dataset 2, and U.K. dataset 3. Analysis was conducted using Python (Python version 3.6 [Python Software Foundation], scikit-learn version 0.24, statsmodels version 0.12). To compensate for dataset enrichment, we rebalanced the datasets when computing the values and CIs for metrics dependent on the prevalence of the subclasses N, S, D, P, H, M, and I. These prevalence-adjusted metrics were the area under the receiver operating characteristic curve, CDR, rule-out rate, false-positive callback reduction rate, and benign-biopsy reduction rate. Metrics for radiologist performance are reported for individual radiologists or for all radiologists in the dataset (collective radiologist performance). For collective radiologist performance, each examination is given equal weight. As a result, radiologists that interpreted more examinations contribute more to the estimate of collective performance.

For sensitivity and CDR metrics, we reported *P* values using a noninferiority *z* test for paired proportions (29) with margins of 5% and 0.25 per 1000 examinations, respectively. For specificity, rule-out rate, and the reduction rates, we computed bootstrap *P* values for a one-sided superiority test through inversion of CI (Appendix S6) (30). A *P* value of .05 was chosen as the threshold for significance.

## Results

### Dataset Demographics

We included female patients from three datasets: U.S. dataset 1 (38 451 patients; mean age, 57.3 years ± 10.0), U.K. dataset 3 (15 025 patients; mean age, 63.5 years ± 7.2), and U.S. dataset 2 (1293 patients; mean age, 55.4 years ± 10.5) (Table 2). The study began with a total of 1 037 543 mammograms. After applying the exclusion criteria, 163 828 examinations from all groups (U.S. dataset 1, 143 593; U.K. dataset 3, 18 873; U.S. dataset 2, 1362) (Fig 1) were included in the final analyses. Race was self-reported by patients. Diagnostic examinations were used only during training to increase the number of cancers available for training (not included in Table 1).

**Table 2:**
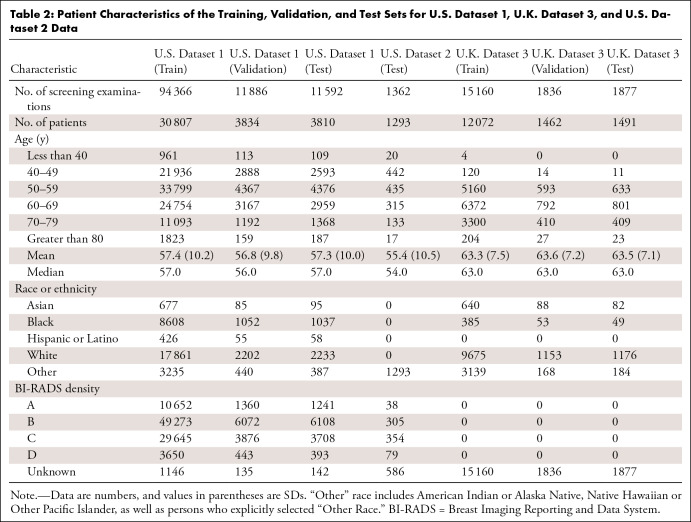
Patient Characteristics of the Training, Validation, and Test Sets for U.S. Dataset 1, U.K. Dataset 3, and U.S. Dataset 2 Data

### Stand-alone Radiologists and Rule-out Device Cancer Detection Performance

**Figure 2:**
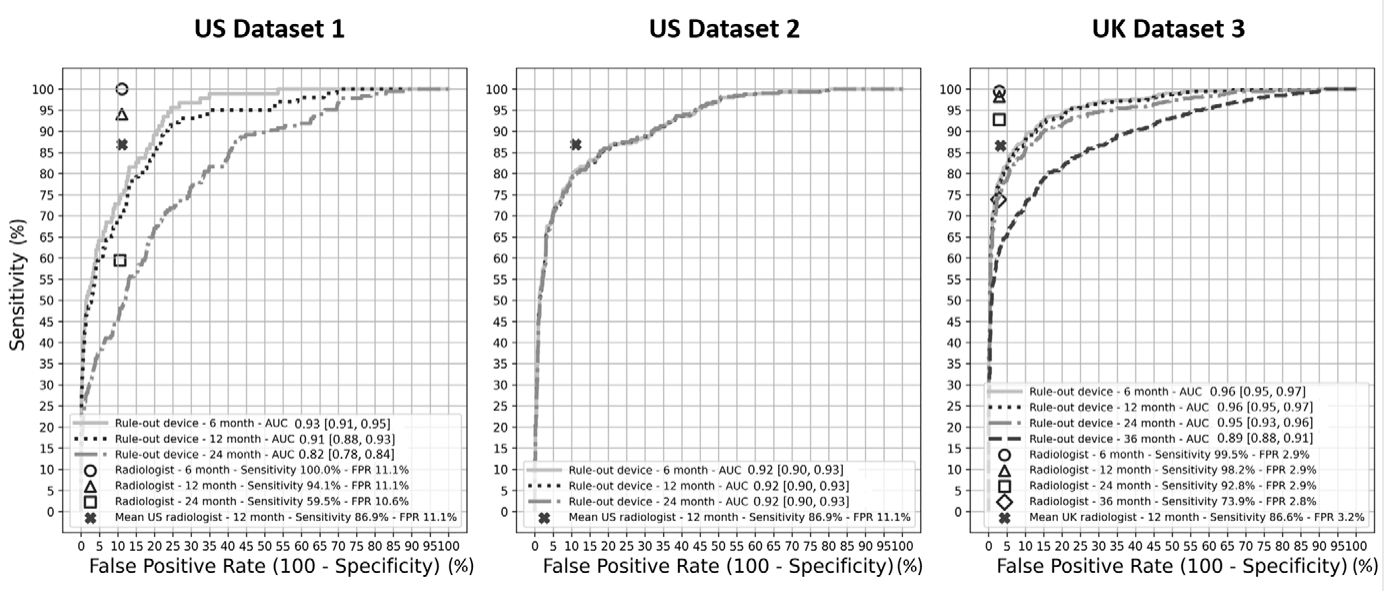
Receiver operating characteristic curves show independent device and radiologist sensitivity and false-positive rates (FPR) calculated for the U.S. and U.K. datasets to predict breast cancer over multiple time windows following a screening examination. The black crosses represent average radiologists’ performance in the United States (Breast Cancer Surveillance Consortium statistics [31]) and the United Kingdom (Cancer Research UK statistics [32]). Hollow marks indicate the average radiologists’ performance as measured in the evaluation study datasets. Area under the receiver operating characteristic curve (AUC) values are presented with 95% CIs in brackets. Since U.S. dataset 2 performs primarily screening examinations, the radiologists’ sensitivity could not be measured as the true extent of false negatives and interval cancers is unknown. The device would perform at a level of performance close to, but not superior to, average radiologists if operated as a stand-alone device at an operating point of balanced sensitivity and specificity. Instead, in this study, the cancer detector is operated as a cancer rule-out device at an extreme operating point on the right side of the receiver operating characteristic curves, with sensitivity nearing 100%.

Figure 2 reports the radiologists’ and rule-out device stand-alone sensitivity and false-positive rates. The receiver operating characteristic curves show that the cancer detection algorithm may be operated as a stand-alone device close to the average performance of radiologists in the United States (31) and United Kingdom (32). Instead, for our simulations of changes in practice, the rule-out device is operated at a point on the right side of the receiver operating characteristic curves, near 100% sensitivity.

### Effect of the Rule-out Device on Quality of Screening for Patients

The main results are for target sensitivities of 99% and 97% (Tables 3, 4). Results are reported for the 12-month and 24-month prediction window for the U.S. and U.K. datasets, respectively. We focused primarily on the 99% sensitivity operating point.

**Table 3:**
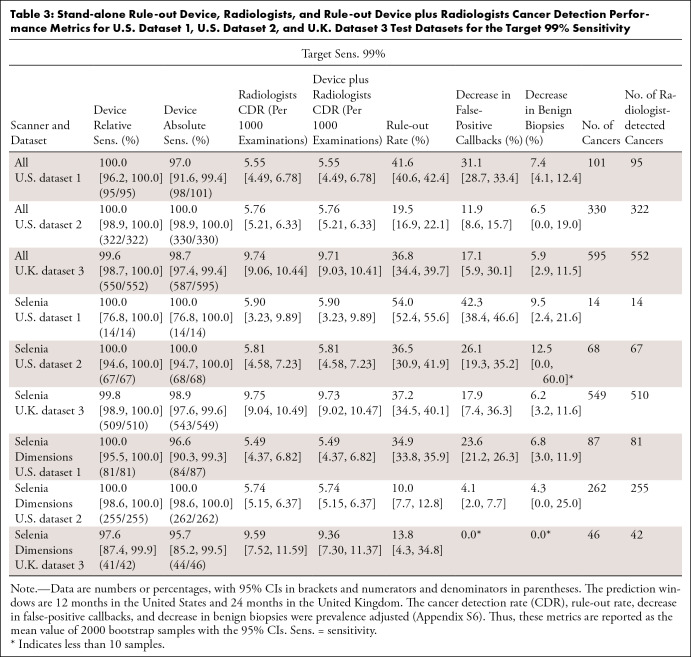
Stand-alone Rule-out Device, Radiologists, and Rule-out Device plus Radiologists Cancer Detection Performance Metrics for U.S. Dataset 1, U.S. Dataset 2, and U.K. Dataset 3 Test Datasets for the Target 99% Sensitivity

**Table 4:**
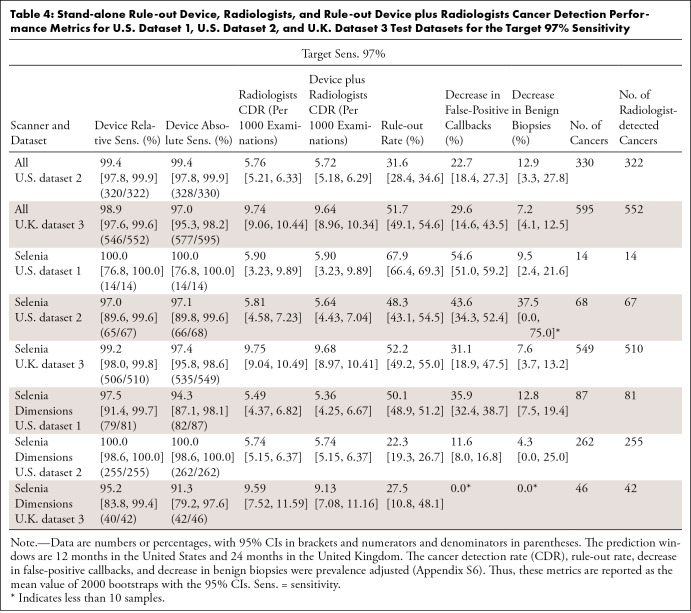
Stand-alone Rule-out Device, Radiologists, and Rule-out Device plus Radiologists Cancer Detection Performance Metrics for U.S. Dataset 1, U.S. Dataset 2, and U.K. Dataset 3 Test Datasets for the Target 97% Sensitivity

At this operating point, the device had a relative sensitivity (the percent of radiologist-detected cancers that were detected by the device) of 100% (95 of 95) in U.S. dataset 1, 100% (322 of 322) in U.S. dataset 2, and 99.6% (550 of 552) in U.K. dataset 3. Two cancers were missed across the three datasets that would have otherwise been detected without the device (Appendix S10). The sensitivity of the screening rule-out workflow (calculated by multiplying the sensitivity of the standard workflow, which is the radiologist sensitivity, and the relative sensitivity of the device) was not inferior to the sensitivity of the standard workflow (5% noninferiority margin: U.S. dataset 1, *P* = .01; U.K. dataset 3, *P* < .001; U.S. dataset 2, *P* < .001). Likewise, the CDR was unaffected by the device, within the noninferiority margin of 0.25 detections per 1000 examinations, in all datasets (U.S. dataset 1, *P* = .02; U.S. dataset 2, *P* < .001; U.K. dataset 3, *P* < .001).

**Figure 3:**
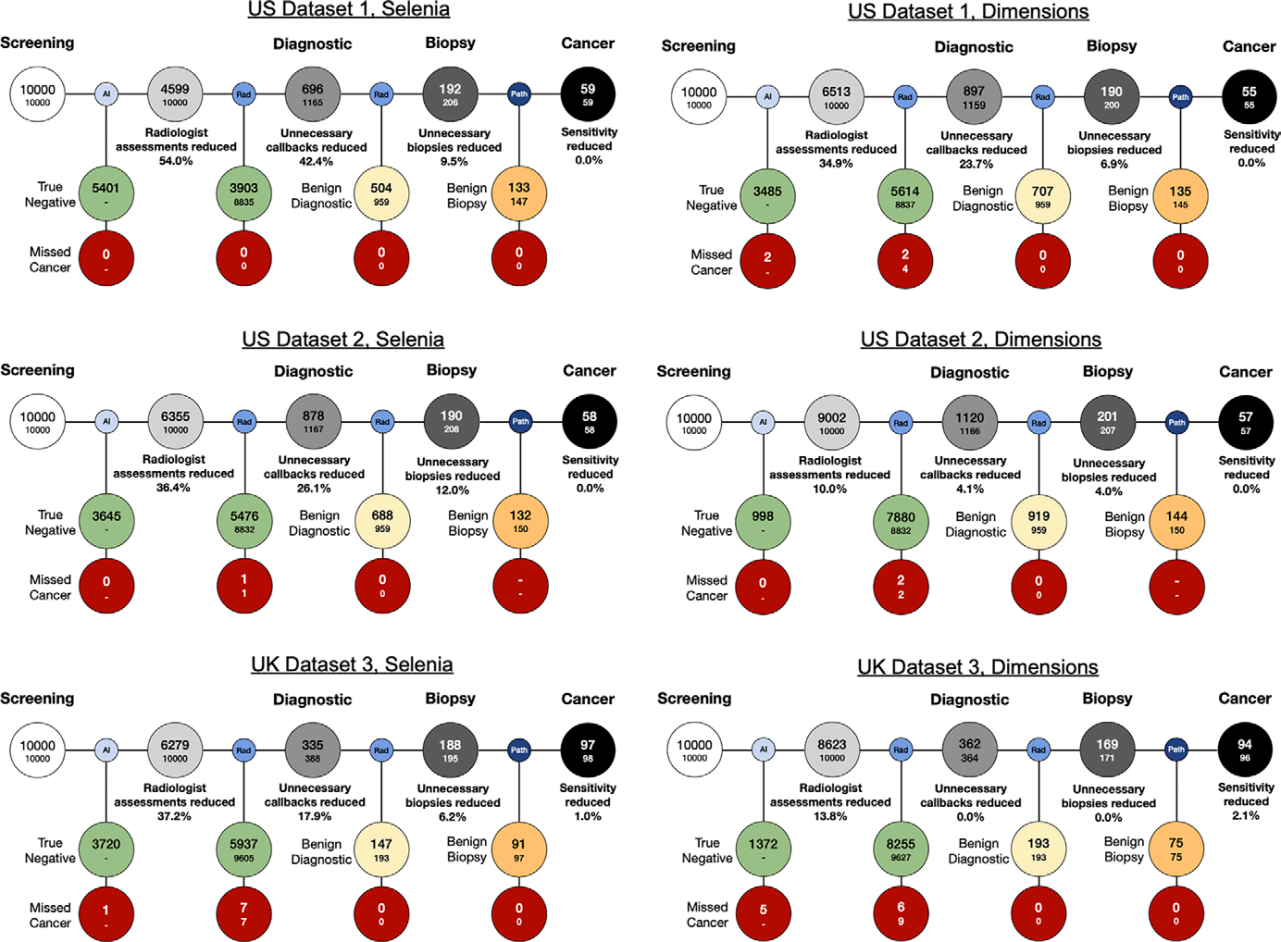
Visualization of the effect of the rule-out device on the screening workflow. The workflow was normalized to 10 000 screening examinations. The rule-out device was operated at a target sensitivity of 99%, and the outcomes were based on information recorded within 12 months from the screening examinations for the United States and 24 months for the United Kingdom. In each node, the small font represents the stand-alone radiologists’ workflow and the large font the rule-out device plus radiologists workflow. By marking a subset of the screening examinations as nonsuspicious, the rule-out device has the downstream effect of reducing false-positive callbacks (reduction greater than 0; U.S. dataset 1, *P* < .001; U.S. dataset 2, *P* < .001; U.K. dataset 3, *P* < .001) and biopsies (reduction greater than 0; U.S. dataset 1, *P* < .001; U.K. dataset 3, *P* < .001; U.S. dataset 2, *P* = .08 [not significant]) while maintaining sensitivity (5% noninferiority margin: U.S. dataset 1, *P* = .01; U.K. dataset 3, *P* < .001; U.S. dataset 2, *P* < .001) (Table 3; Appendix S6).

The device marked the following (prevalence-adjusted) percentages of mammograms as nonsuspicious: 41.6% (95% CI: 40.6%, 42.4%) in U.S. dataset 1, 19.5% (95% CI: 16.9%, 22.1%) in U.S. dataset 2, and 36.8% (95% CI: 34.4%, 39.7%) in U.K. dataset 3. The device also marked several clinical false positives as nonsuspicious, reducing false-positive callbacks by 31.1% (95% CI: 28.7%, 33.4%) in U.S. dataset 1, 11.9% (95% CI: 8.6%, 15.7%) in U.S. dataset 2, and 17.1% (95% CI: 5.9%, 30.1%) in U.K. dataset 3. These reduction rates were significantly larger than 0 (U.S. dataset 1,* P* < .001; U.S. dataset 2,* P* < .001; U.K. dataset 3, *P* < .001). Similarly, the device reduced the number of negative biopsies by 7.4% (95% CI: 4.1%, 12.4%) in U.S. dataset 1, 6.5% (95% CI: 0.0%, 19.0%) in U.S. dataset 2, and 5.9% (95% CI: 2.9%, 11.5%) in U.K. dataset 3. These reduction rates were significantly larger than 0 for U.S. dataset 1 and U.K. dataset 3 (U.S. dataset 1,* P* < .001; U.K. dataset 3, *P* < .001; U.S. dataset 2,* P* = .08) (Fig 3).

We also stratified the results according to the scanner models (HSE and SED) used. For the target 99% sensitivity, HSE examinations had a higher relative and absolute sensitivity, rule-out rate, reduction of false-positive callbacks, and reduction of benign biopsies than SED examinations. The differences in per-scanner performance were likely attributable to the differences in the number of cancer-positive mammograms in the development dataset, 4867 for scanner model HSE and 1294 for SED (Appendices S1 and S11).

### Effect of the Rule-out Device on Radiologists’ Performance

**Figure 4:**
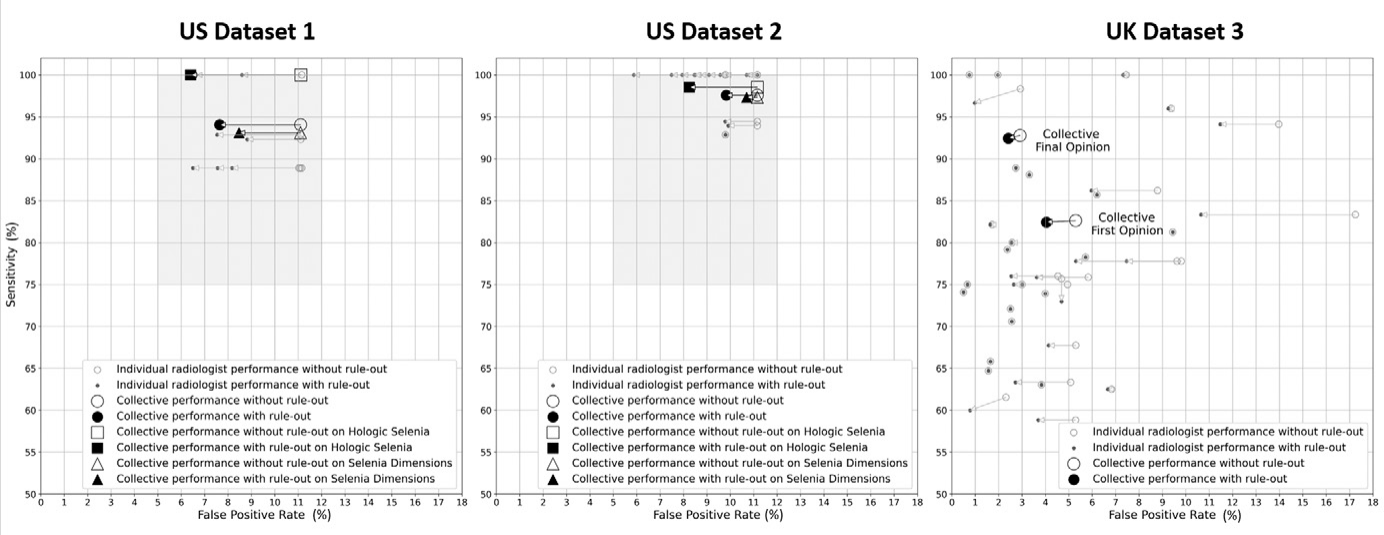
Graphs show individual and collective radiologists’ sensitivity and false-positive rates with and without the rule-out device. Small circular marks report the effect of the rule-out device on the cancer detection performance of individual radiologists. Large circular marks report the effect on their collective performance. In the U.S. dataset 1 and U.S. dataset 2 results, large square and triangular marks report the effect of the rule-out device on radiologists’ collective performance when reading examinations from the Hologic Selenia and Hologic Selenia Dimensions scanners, respectively. This breakdown is not reported for U.K. dataset 3 as it consists primarily of examinations acquired with the Hologic Selenia (95.9%). In the U.K. paradigm with multiple readers, the collective first opinion refers to the decision of the first reader, while the collective final opinion refers to the final decision based on radiologists’ consensus. The performance of an individual radiologist is based on all opinions provided by the radiologist, as the first, second, or arbitrating reader. In the U.S. results, the gray rectangle represents the region of acceptable performance as defined by Lehman et al (31). The U.K. National Health System considers acceptable false-positive rates between 3% and 9% and does not define explicit thresholds of acceptable performance for sensitivity, focusing instead on performance thresholds for the cancer detection rate (33). Outcomes are derived from examinations within 12 months from the screening examination for the United States and within 24 months for the United Kingdom. Radiologists were included in this analysis if they read at least 10 cancer-positive and 10 cancer-negative screening examinations within this study dataset.

We evaluated the potential effect of the device on the individual radiologists and on their collective performance. This analysis included both scanner models. Figure 4 compares individual and collective radiologists’ sensitivity and false-positive rates with and without the device. For the double-reading system in U.K. dataset 3, the device improved specificity from 94.7% to 96.0% (95% CI: 94.6%, 96.7%) for the first reader (*P* = .02) and from 97.1% to 97.6% (95% CI: 97.2%, 98.0%) for the last reader (*P* = .01). Sensitivity was noninferior, changing from 82.6% (492 of 595) (95% CI: 79.1%, 85.8%) to 82.4% (490 of 595) (95% CI: 78.9%, 85.6%) for the first reader (*P* < .001) and from 92.8% (552 of 595) (95% CI: 90.0%, 94.6%) to 92.4% (550 of 595) (95% CI: 89.6%, 94.3%) (*P* < .001) for the last reader. For the single-reader system in U.S. dataset 1, the device had a more pronounced effect. The average U.S. dataset 1 radiologist's specificity increased (*P* < .001) from 88.9% to 92.4% (95% CI: 92.1%, 92.7%) while maintaining sensitivity (*P* = .01 with 5% noninferiority margin) at 94.1% (95 of 101) (95% CI: 87.5%, 97.8%). For U.S. dataset 2, the rule-out device increased specificity (*P* < .001) from 88.8% to 90.2% (95% CI: 89.8%, 90.6%) and maintained sensitivity at 97.6% (322 of 330) (95% CI: 95.3%, 99.0%) (*P* < .001 with 5% noninferiority margin).

## Discussion

This work demonstrates a rule-out device operating at high sensitivity can potentially reduce the number of screening examinations requiring radiologist interpretation by nearly 41.6% (95% CI: 40.6%, 42.4%; *P* < .001). This device can also potentially reduce false-positive callbacks by 31.1% (95% CI: 28.7%, 33.4%; *P* < .001) and benign biopsies by 7.4% (95% CI: 4.1%, 12.4%; *P* < .001). While previous works have shown a reduction in benign biopsies given localizations from radiologists (34), to our knowledge, this is the first work that has shown a fully autonomous reduction in benign biopsies in simulations. To accomplish this, we introduced a labeling scheme that categorized examinations as malignant, high risk, pathology benign, diagnostic benign, screening benign, and negative to model the clinical pathway. Our stratification analysis, not analyzed by previous works, revealed that performance differs by scanner model, an important facet to consider during evaluation. Overall, while our device's performance was lower than the radiologists at a similar operating point, the rule-out device is designed to complement the radiologist and operate at a point of high sensitivity, leading to improvements for the potential human plus AI paradigm.

Reduced false positives may increase screening compliance as false positives and anxiety are linked to lower screening compliance rates (14). This reduction also prevents the financial burden of follow-up examinations and treatment. Shortages of health care providers, including radiologists and technologists, limit patient access. Lower utilization of diagnostic and biopsy studies could provide patients with more prompt access to definitive diagnoses. Lastly, reducing radiologists’ workloads can also mitigate burnout, address workforce shortages, and help expand nascent under-resourced screening systems. Currently, to address false positives, the USPSTF biennial screening recommendations (to those over 50 years of age) were estimated to reduce false positives by 68% at the cost of reducing sensitivity and the number of deaths averted by 30% (16). Consequently, many individuals would die of breast cancers that would have otherwise been found by more frequent screening. However, our study showed that algorithms may achieve at least half of the reduction of the false-positive rate achieved by the USPSTF guidelines update while reducing the sensitivity by only 1%.

We performed stratification analysis to reveal differences in subset performance. For a given dataset, the two scanner models had differing performances. Therefore, both the dataset and scanner models are interpreted differently by the model. Additionally, when selecting our operating point, we targeted 99% and 97% sensitivity from the validation set. The SED examinations had lower rule-out, false-positive reduction, and benign biopsy reduction rates than HSE examinations. Instead, evaluating SED examinations at the 97% target sensitivity was more comparable to HSE examinations at the target sensitivity of 99%. This pattern was also mirrored when comparing U.S. dataset 2 and U.K. dataset 3 at the 97% target sensitivity to U.S. dataset 1 at the 99% target sensitivity. These findings suggest the possible need for selection of the operating point based on the site and scanner model.

Evaluating the clinical workflow, the device may affect individual radiologist performance, reducing the false-positive rate without substantially reducing the sensitivity. Our data show that the device has the strongest effect on radiologists with high false-positive rates. Our analysis also introduces the concept of relative sensitivity, meaning that the device still detects the cancers that radiologists detect. We observe that even when the absolute sensitivity is 97.0%, the relative sensitivity can remain at 100%, suggesting that no new cancers would be lost in this paradigm.

There were limitations to this study. Our evaluation is a retrospective simulation that assumes that radiologist behavior is unaffected by the removal of examinations from their worklist. Thus, reader studies are required to further investigate the impact of rule-out on radiologist behavior and workflows. The test sets, particularly that of U.S. dataset 1, included multiple examinations of some patients. As a result, the variance of different metrics could be underestimated due to the correlation between different examinations of the same patient. Also, evaluation over all scanners in the dataset elicits a performance higher than that of SED but lower than that of HSE. The cancer cases with SED scanners were limited in training and testing (except for the U.S. dataset 2 testing dataset), and there was about one-fourth of the number of cancer cases with HSE scanners. Thus, the performance can appear better when not stratified on the scanner model, an issue that may have caused past studies to overestimate performance in real-world settings. Additionally, false-negative information for U.S. dataset 2 data was limited as we did not have many years of examinations like we did for U.S. dataset 1 and U.K. dataset 3. Thus, the device absolute sensitivity and sensitivity of radiologists were both abnormally high (Appendix S1). Finally, radiologists’ false negatives were defined without discerning between missed cancers and true interval cancers not visible at the examination. Also, these false negatives may not be tracked by the clinics where the examinations were performed.

In conclusion, rule-out devices promise to have several benefits. The elimination of incorrect follow-up examinations and biopsies, which constitute major limitations of breast cancer screening today, benefits patients directly and is the most critical advantage of cancer rule-out technology. Quality assurance and monitoring systems must be devised to guarantee the safe operation of rule-out devices, and further investigations are required to substantiate the benefits to patients, radiologists, and the health care system. With these measures in place, rule-out devices could offer a safer and more effective alternative to improving screening than restrictive nationwide guideline changes.
